# Cystic Fibrosis: A Disease with a New Face

**DOI:** 10.3390/life16020228

**Published:** 2026-01-30

**Authors:** Janice Wang, Patricia A. Walker

**Affiliations:** 1 Division of Pulmonary, Critical Care and Sleep Medicine, Long Island Jewish Medical Center, Donald and Barbara Zucker School of Medicine at Hofstra/Northwell, New York, NY 11042, USA; 2 Division of Pulmonary, Critical Care and Sleep Medicine, Lenox Hill Hospital, Donald and Barbara Zucker School of Medicine at Hofstra/Northwell, New York, NY 10075, USA

Medical advancements in the field of Cystic Fibrosis (CF), a rare, genetic disorder, have been profound over the last decade, allowing care to evolve in ways previously thought unimaginable by those living with CF and those caring for people with CF.

Once uniformly fatal in childhood, CF has become a chronic, increasingly adult disease characterized by extended survival, evolving clinical phenotypes, and unprecedented therapeutic opportunity.

Over the past several decades, the establishment of specialized CF centers providing multidisciplinary care, aggressive management of airway infection and inflammation, and improved nutritional support have steadily improved outcomes. However, no development has altered the trajectory of CF as fundamentally as the advent of highly effective cystic fibrosis transmembrane conductance regulator (CFTR) modulator therapies.

The identification of the CFTR gene more than three decades ago provided the first molecular explanation for CF and laid the foundation for targeted therapy. Clinical care, however, remained focused on mitigating downstream consequences of CFTR dysfunction—airway infection, bronchiectasis, malnutrition, and progressive organ failure.

The introduction of CFTR modulators, particularly the combination of elexacaftor/tezacaftor/ivacaftor (ETI), marked a turning point. For most individuals with CF, these therapies substantially improve lung function, nutritional status, quality of life, and survival. As a result, CF is now increasingly recognized as a lifelong condition with a growing adult population and a shifting spectrum of disease manifestations.

Although most of the population in the U.S. with CF benefit from CFTR modulators, 6% of people remain with CF without a treatment targeting their specific genetic mutations [1]. Furthermore, worldwide, not all individuals with CF have the option for or access to highly effective modulator therapies.

The impact of this therapeutic revolution is reflected in contemporary survival estimates. Today, the median predicted survival for individuals born with CF in the United States approaches 65 years. These gains represent one of the most striking success stories in genetic medicine. At the same time, they introduce new challenges: the emergence of aging-related comorbidities, evolving reproductive and family planning needs, long-term medication effects, and the need to redefine what optimal CF care means in the era of disease modification.

Despite the remarkable success of CFTR modulators, CF has not been “cured”. For those who do not have a modulator, or who do not respond, many challenges exist. Structural lung disease, once established, does not fully reverse, and chronic complications such as CF-related diabetes (CFRD), liver disease, bone disease, and malignancies continue to affect long-term outcomes. Moreover, as CF phenotypes become more heterogeneous and milder forms of CFTR dysfunction are increasingly recognized, diagnosis itself has become more complex. CF is no longer solely a pediatric disease with a typical presentation. It is now frequently diagnosed in adulthood, and often in individuals with atypical or organ specific manifestations.

In this Special Issue of 16 manuscripts, “Cystic Fibrosis: A Disease with a New Face”, we visit the past, review the present, and anticipate the future of CF as the medical and scientific fields of this orphan disease make remarkable advancements one would hope to see with every disease. We explore aspects of CF care that have transformed over time, with eight review articles evaluating newborn screening and diagnostic challenges in all ages, lung transplant, future therapeutics, comorbidities of an aging population, symptom burden, and palliative care; quality of life and well-being, reproductive medical care, nutritional care guidelines, and less studied in people with CF, but equally important, disordered eating behaviors [2,3,4,5,6,7,8,9].

In eight additional articles, original investigations demonstrate and reaffirm insights into the context of HEMT. Real-world data were used to evaluate the prevalence of CF-related diabetes (CFRD), adverse events, and drug-drug interactions with elexacaftor/tezacaftor/ivacaftor (ETI), providing valuable insight into the clinical impact of ETI since its commercial availability [10,11]. Three articles highlight how ETI-associated improvement is exhibited in areas of CF-associated liver disease, glycemic control in CFRD, and nocturnal oxygenation and lung function [12,13,14]. Appreciation for the complexities of CF as a highly variable phenotypic disease with the inheritance of complex alleles in Mexican families is shown in a featured article by Mendiola-Vidal et al. [15]. However, knowledge gaps remain, given the complex pathogenicity of CF, and are explored in studies evaluating serum levels of calcitonin gene-related peptide (CGRP) levels in people with CF and HEMT-restorative effects on pancreatic ductal epithelial cells in CF [16,17].

Together, these manuscripts reinforce a central theme: CF has become a biologically and clinically diverse disease in which traditional categories are increasingly inadequate. Modulator therapy has narrowed but not eliminated the gap between genotype and phenotype. As individuals with CF live longer and healthier lives, the full spectrum of CFTR dysfunction, from classic CF to CFTR-related disorders, becomes more visible. This complexity demands a more nuanced approach to diagnosis, long-term management, and research prioritization.

Looking forward, the future of CF therapy is both promising and demanding. Gene-based and mutation-agnostic therapies, including mRNA delivery, gene editing, and novel CFTR correction strategies, hold the potential to address the unmet needs of individuals who do not benefit from current modulators. At the same time, there is an increasing need for long-term observational data to define the durability, safety, and broader health effects of CFTR modulators across decades of use.

The growing adult CF population requires expanded expertise in managing metabolic, hepatic, oncologic, and reproductive conditions, as well as innovative models of care that support transitions across the lifespan.

The transformation of CF also carries implications beyond the disease itself. More than ever before, CF has become a paradigm for precision medicine, demonstrating how molecular diagnosis, targeted therapy, and comprehensive clinical infrastructure can converge to change the natural history of a genetic disease. However, this success also highlights the importance of equitable access, global implementation, and sustained investment in research to ensure that all individuals with CF benefit from ongoing scientific advances.

This Special Issue captures CF at a moment of extraordinary transition. The disease that once defined pediatric pulmonary medicine is now a complex, lifelong condition shaped by powerful therapies, diverse genetic and phenotypic expression, and expanding clinical horizons.

The articles presented here not only document how far the field has come but also illuminate the challenges and opportunities that lie ahead. As CF continues to take on a new face, continued innovation, careful study, and collaborative care will be essential to ensure that the promise of this new era is fully realized for all who live with this disease.
